# Maintenance Treatment With Low-Dose Decitabine After Allogeneic Hematopoietic Cell Transplantation in Patients With Adult Acute Lymphoblastic Leukemia

**DOI:** 10.3389/fonc.2021.710545

**Published:** 2021-08-16

**Authors:** Jia Liu, Zhong-Xing Jiang, Xin-Sheng Xie, Ding-Ming Wan, Wei-Jie Cao, Meng Wang, Zhen-Zhen Liu, Zhen-Kun Dong, Hai-Qiong Wang, Run-Qing Lu, Yin-Yin Zhang, Qian-Qian Cheng, Ji-Xin Fan, Wei Li, Fei He, Rong Guo

**Affiliations:** ^1^Department of Hematology, The First Affiliated Hospital of Zhengzhou University, Zhengzhou, China; ^2^Hematopoietic Stem Cell Transplantation Center, Institute of Hematology and Blood Diseases Hospital, Chinese Academy of Medical Sciences and Peking Union Medical College, Tianjin, China; ^3^Department of Cardiology, The First Affiliated Hospital of Zhengzhou University, Zhengzhou, China

**Keywords:** allogeneic hematopoietic stem cell transplantation, decitabine, maintenance, prophylaxis, relapse, acute lymphoblastic leukemia

## Abstract

**Background:**

Post-transplant relapse remains a principal leading cause of failure after allogeneic hematopoietic stem cell transplantation (allo-HSCT) in patients with adult acute lymphoblastic leukemia (ALL). The aim of this study was to investigate the efficacy and safety of low-dose decitabine on the prevention of adult ALL relapse after allo-HSCT.

**Methods:**

In this prospective study, we enrolled 34 patients with ALL who underwent allo-HSCT from August 2016 to April 2020 and received low-dose decitabine maintenance treatment after transplantation. The primary objectives were cumulative incidence of relapse rate (CIR), overall survival (OS), and disease-free survival (DFS). The secondary objectives were graft-*versus*-host disease (GVHD) and safety.

**Results:**

Among the enrolled 34 patients, 6 patients relapsed and 6 patients died. The 2-year CIR, OS, and DFS were 20.2, 77.5, and 73.6%, respectively. Subgroup analysis revealed the 2-year CIR, OS, and DFS rates of 12 patients with T-ALL/lymphoblastic lymphoma (LBL) were 8.3, 90, and 81.5%, respectively. None of the seven patients with T-ALL relapsed. During maintenance treatment, only one patient (2.9%) developed grade IV acute GVHD and four (11.8%) patients had severe chronic GVHD. Thirty-two patients (94.1%) developed only grade I to II myelosuppression, and two patients (5.8%) developed grade III to IV granulocytopenia.

**Conclusions:**

Maintenance treatment with low-dose decitabine after allo-HSCT may be used as a therapeutic option to reduce relapse in patients with adult ALL, especially in patients with T-ALL. Our findings require confirmation in larger-scale controlled trials.

**Clinical Trial Registration:**

Chinese Clinical Trials Registry, identifier ChiCTR1800014888.

## Introduction

Post-transplant relapse remains a leading cause of failure after allogeneic hematopoietic stem cell transplantation (allo-HSCT). In patients with adult acute lymphoblastic leukemia (ALL), the risk of relapse-related death is higher, up to 30–54% (1–3). At present, donor lymphocyte infusion (DLI) is the most widely used management approach to relapse after transplantation. However, the downregulation of human leukocyte antigen II (HLA-II) molecules leads to the inability of donor T cells to recognize leukemic cells, which limits the use of DLI in the treatment of relapse after transplantation in patients with acute myeloid leukemia (AML) (4, 5), and the 3-year overall survival (OS) rate of these patients is only 10–20% (6). In particular, DLI is not ideal for treatment of ALL relapse after transplantation. Given the difficulty in the treatment of post-transplant relapse, preventing relapse is more important than treatment. Therefore, it is urgent to explore novel approaches to prevent leukemia relapse after allo-HSCT in adult ALL.

Different from the relapse occurring after traditional chemotherapy, the elimination of leukemic cells after allo-HSCT mainly depends on the graft-*versus*-leukemia (GVL) effect (7). A mechanism of post-transplantation relapse involves the downregulation of HLA-class II molecules induced by epigenetic silencing to reduce the GVL effect, and the downregulation of HLA-II expression is caused by hypermethylation of the promoter of the class II major histocompatibility complex (MHC) transactivator (CIITA) (4, 5, 8). Most patients with T-ALL showed molecular loss of HLA-II (9), and only 5–17% of T-ALL expressed HLA-DR. A similar mechanism of loss of expression of HLA-II class molecules was also observed in B-cell lymphoma lines (10). In addition, many studies have shown that the degree of methylation of tumor suppressor genes is closely associated with the subtypes and prognosis of ALL (11–13). The above studies indicate the possibility of using hypomethylating agents (HMAs) as treatment in ALL after transplantation.

Both decitabine and azacitidine are HMAs and have been safely and effectively used for maintenance treatment of AML and myelodysplastic syndrome (MDS) after transplantation (14–20). The main effect of post-transplantation hypomethylation treatment is to prevent primary disease relapse and reduce graft-*versus*-host disease (GVHD). The main mechanisms include increasing the number of regulatory T (Treg) cells and inducing cytotoxic CD8+ T cells (21, 22). Thus, considering the low hematological toxicity of maintenance treatment with low-dose decitabine after AML/MDS transplantation and the advantages of preventing relapse without affecting GVHD, we administered low-dose decitabine maintenance treatment to 34 patients with ALL after allo-HSCT. This was the first prospective study with the largest number of cases to date to describe the application of decitabine prophylaxis for relapse of transplanted ALL.

## Materials and Methods

### Study Design

This was a single-center, prospective, single-arm study. Informed consent was obtained from all patients, and the study was conducted in accordance with the Declaration of Helsinki. The study protocol was approved by the Ethics Committee of the First Affiliated Hospital of Zhengzhou University. This study is registered at www.chictr.org.cn (ChiCTR1800014888).

### Patient Cohort

Eligible candidates met all of the following inclusion criteria: (1) age ≥14 years; (2) satisfied the diagnostic criteria of ALL or lymphoblastic lymphoma (LBL) in accordance with WHO 2016 guidelines (22); (3) patients underwent allo-HSCT in the First Affiliated Hospital of Zhengzhou University; (4) Eastern Cooperative Oncology Group (ECOG) performance status score ≤2; (5) morphological complete remission (CR) before maintenance treatment; (6) estimated survival ≥3 months.

The exclusion criteria included (1) concomitant diagnosis of another cancer; (2) concomitant uncontrolled fungal, bacterial, or viral infection; (3) hypersensitivity to decitabine; (4) diagnosis of human immunodeficiency virus infection or in active stage of hepatitis B or C virus infection; (5) brain dysfunction or severe mental illness; (6) concomitant disease(s) that may seriously endanger the safety of patients or affect the completion of this study; and (7) participation in another drug clinical trial(s) 1 month before the trial.

### Maintenance Treatment Regimen

Maintenance treatment began more than 50 days after transplantation. This post-HSCT interval allows for adequate marrow recovery before starting decitabine. Decitabine 10 mg/d was planned for intravenous infusion 5 h on days 1 to 5, and every 4 weeks for eight cycles, based on comprehensive analysis of previously relevant studies (15, 23, 24). However, in the previous pretrial of low-dose decitabine maintenance treatment after AML/MDS transplantation at our center, patients who received decitabine for 5 days at 10 mg/d developed grade IV myelosuppression with granulocytic fever, requiring transfusion of approximately two units of platelets. Myelosuppression was alleviated, and blood products were not needed after adjusting to 10 mg/d for 3 days. Therefore, decitabine 10 mg/d (approximately 6 mg/m^2^/d) was ultimately administered as an intravenous infusion for 5 h on days 1, 3, and 5 every 4 weeks for eight cycles in this study. It should be noted that the number of cycles increased by four to six cycles based on the patients’ wishes, if they presented minimal residual disease (MRD) in the late period of maintenance treatment. The interval time of each cycle was also appropriately prolonged according to the recovery of the patient’s hemogram.

Routine blood parameters, bone marrow (BM) smear, and MRD were examined before each cycle. MRD detection methods included flow cytometry (FCM), quantitative detection of certain genes or WT1 *via* polymerase chain reaction (PCR), and donor chimerism. In addition, patients with T-LBL also underwent regular positron emission tomography-computed tomography (PET/CT) examinations. Routine blood parameters were examined intermittently during the period of drug administration. Granulocyte colony stimulating factor (G-CSF) or blood products were administered as required according to the hemogram. Systemic anticancer drugs and other similar experimental treatments were banned during the trial. Withdrawal criteria included (1) patients who were unable to tolerate the treatment, (2) patients with relapse of primary disease, (3) patients developing severe GVHD or unacceptable infection, and (4) subjects who decided to withdraw from the trial.

### Evaluation Parameters

Patients with ALL were divided into high-risk and standard-risk groups. High-risk ALL was defined based on at least one of the following criteria: (1) age ≥35 years; (2) white blood cell (WBC) counts >30×10^9^/L for B-cell precursor (BCP)-ALL or >100×10^9^/L for thymic T-ALL; (3) pro-B-ALL (CD10−), early T-ALL or mature T-ALL, hypodiploid ALL; (4) ALL with Philadelphia chromosome (Ph), with the t(4,11) translocation, or with complex karyotype; and (5) failure to achieve CR after the first induction therapy (25). The risk classification of LBL was based on the international prognostic index (IPI) score. CR from ALL was defined as BM blasts <5%, no primitive naive lymphocytes in the peripheral blood, and no extramedullary lesions. CR of LBL was defined as PET/CT with no positive lesions and a normal BM smear. MRD-positive was defined as FCM >0.01% of cells with a leukemia-associated aberrant immune phenotype in the BM sample or BCR-ABL transcript level >0% in patients with Ph+ ALL. Acute GVHD (aGVHD) and chronic GVHD (cGVHD) were graded according to accepted international criteria (26, 27). Considering that the platelet count was lower than the normal value and the WBC count was normal in some patients before treatment, hematological adverse reactions after treatment were evaluated based on changes in WBCs and were graded according to the National Cancer Institute Common Toxicity Criteria, version 3.0.

### Statistical Analysis

The follow-up deadline was July 31, 2020. The primary endpoints were cumulative incidence of relapse rate (CIR), overall survival (OS), and disease-free survival (DFS) of patients who received low-dose decitabine maintenance treatment. The secondary endpoints were the incidence of GVHD after receiving decitabine and the safety of low-dose decitabine maintenance regimen. Statistical analyses were performed using SPSS software (version 21.0), R software package (version 4.0.0), and GraphPad Prism (version 8.0). Descriptive statistics were used to describe the general clinical features of patients. Data were censored at the time of relapse, non-relapse mortality (NRM), or last available follow-up. The cumulative incidence of relapse (CIR) and NRM were performed using the competing risk model, in which death without relapse was considered a competing risk of relapse. The disease-free survival (DFS) and OS were calculated using the Kaplan-Meier method. CIR was defined as time from transplantation to relapse. OS was defined as the time from transplantation to death from any cause. DFS was defined as time from transplantation to relapse or death, whichever occurred first. Statistical significance was defined as *P* < 0.05.

## Results

### Patient Characteristics

In total, 34 patients from our institution were enrolled between August 2016 and April 2020. The characteristics of patients are shown in **Table 1**. Our cohort comprised 34 patients with a median age of 20 years (range, 14–49 years), including 22 males and 12 females. Overall, 22 patients (64.7%) had B-ALL, 7 (20.6%) had T-ALL, and 5 (14.7%) had T-LBL. Nine patients (26.5%) were at standard-risk, and 25 (73.5%) were at high-risk (including five patients with T-LBL). Seven patients (20.6%) were Philadelphia chromosome-positive (Ph^+^), and 27 patients (79.4%) were Philadelphia chromosome-negative (Ph^−^). Excluding six patients due to missing MRD data from other hospitals at initial treatment, 10 (35.7%) of 28 assessable patients were MRD positive (including two patients with non-CR after induction) and 18 patients (64.3%) who became MRD-negative after the first induction chemotherapy. Seven patients (20.6%) became MRD-positive, and 27 patients (79.4%) achieved MRD negativity at transplantation. All patients received myeloablative conditioning, including 26 patients (76.5%) receiving a modified busulfan (Bu)/cyclophosphamide (Cy) regimen and 8 (23.5%) patients receiving a total body irradiation (TBI)/Cy regimen (26). Prophylaxis against GVHD for all patients consisted of cyclosporine A and short-term methotrexate treatment with mycophenolate mofetil. In addition, patients without matched related donors were supplemented with anti-thymocyte globulin. The median number of infused CD34+ cells was 5.7 × 10^6^/kg (range, 1.52–16.3 × 10^6^/kg), and the median number of infused MNC cells was 5.3 × 10^8^/kg (range, 1.2–11.5 × 10^8^/kg). Neutrophils and platelets were implanted successfully in all patients. All patients achieved morphological CR and donor complete chimerism before maintenance treatment. Thirty patients (88.3%) achieved MRD negativity, and four patients (11.7%) were MRD positive before maintenance treatment (**Table 2**).

**Table 1:** Patients’ characteristics (N = 34).

Characteristic	Value
Age at HSCT, year, range (median)	15–49 (20)
Sex, n (%)	
Male	22 (64.7)
Female	12 (35.3)
Diagnosis, n (%)	
B-ALL	22 (64.7)
T-ALL/T-LBL	7/5 (35.3)
Risk classification, n (%)	
High risk	25 (73.5)
Standard risk	9 (26.5)
Subtype, n (%) some-positive (Ph+) ALL	
Ph^+^ ALL	7 (20.6)
Ph^−^ ALL	27 (79.4)
MRD after the 1^st^ induction, n (%)	
Negative	18 (64.3)
Positive	10 (35.7)
MRD at allo-HSCT, n (%)	
Negative	27 (79.4)
Positive	7 (20.6)
Disease status at allo-HSCT, n (%)	
CR1	31 (91.2)
CR2	3 (8.8)
HCI-CI score, n (%)	
0	21 (61.8)
1	12 (35.3)
2	1 (2.9)
EBMT risk score, n (%)	
0	4 (11.8)
1–2	25 (73.5)
3–4	5 (2.9)
Conditioning regimen, n (%)	
mBu/Cy	26 (76.5)
TBI/Cy	8 (23.5)
Transplant resource, n (%)	
PBSC	31 (91.2)
PBSC+BM	3 (8.8)
Donor/HLA match, n (%)	
Matched related	21 (61.8)
Mismatched related	11 (32.4)
Matched unrelated	1 (2.9)
Mismatched unrelated	1 (2.9)
CD34^+^ cells×10^6^/kg, range (median)	1.52–16.3 (5.7)
MNC cells×10^8^/kg, range (median)	1.2–11.5 (5.3)
Time of leukocyte engraftment, d (median)	8–21 (13)
Time of platelet engraftment, d (median)	10–22 (14)

ALL, acute lymphoblastic leukemia; LBL, lymphoblastic lymphoma; Ph, Philadelphia chromosome; MRD, minimal residual disease; HCT-CI, hematopoietic cell transplantation comorbidity index; EBMT, European Society for Blood and Marrow Transplantation; Bu, busulfan; Cy, cyclophosphamide; TBI, total body irradiation; mBu/Cy, modified Bu/Cy; PBSC, peripheral blood stem cell; BM, bone marrow; MNC, mononuclear cell.

**Table 2:** Outcomes of transplantation and maintenance treatment (N = 34).

Outcomes	Data
MRD before maintenance treatment, n (%)	
Positive	4 (11.7)
Negative	30 (88.3)
Start time of decitabine, d, median (range)	96 (51–175)
No. of cycles, median (range)	7 (1–14)
Completed study, n (%)	14 (41.1)
Maintenance period, n (%)	8 (23.5)
Reason for discontinuation, n (%)	
Withdrew consent	5 (14.7)
Relapse	4 (11.7)
GVHD	3 (8.8)
Hematological toxicity, n (%)	
I∼II	32 (94.1)
III∼IV	2 (5.8)
Acute GVHD after maintenance treatment, n (%)	1 (2.9)
I∼II	0
III∼IV	1 (2.9)
Chronic GVHD after maintenance treatment, n (%) nnnn(%), n(%)n(%)	7 (20.5)
Mild	3 (8.8)
Moderate	0
Severe	4 (11.8)
Relapse, n (%)	6 (17.6)
B-ALL	5 (14.7)
T-LBL	1 (2.9)
Cause of death, n (%)	6 (17.6)
Relapse	4 (11.7)
Infection	1 (2.9)
GVHD	1 (2.9)
Duration of follow-up, d, range (median)	154–1,629 (480.5)

GVHD, graft-versus-host disease.

### Decitabine Exposure and MRD

**Figure 1:**
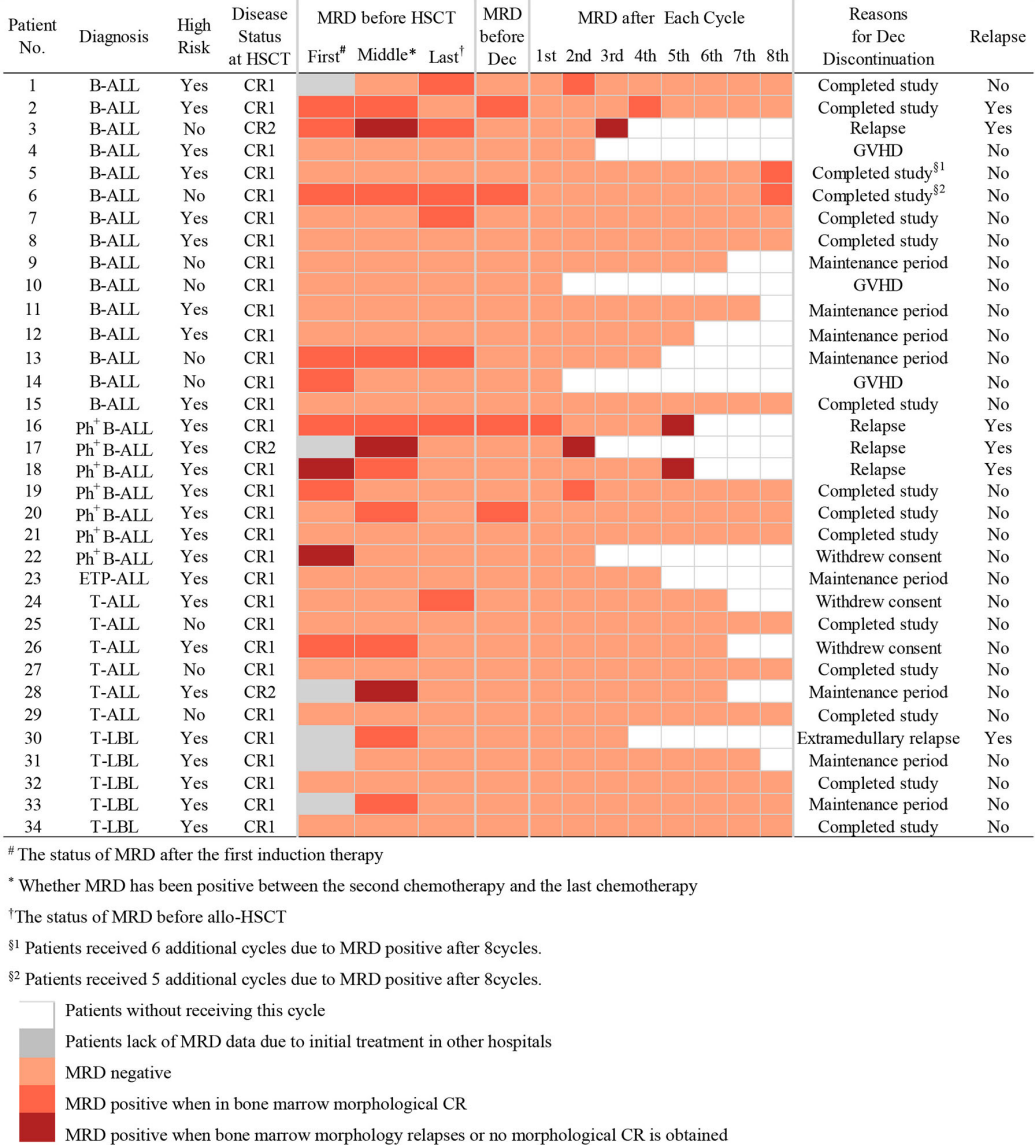
Changes in MDR and decitabine exposure in patients.

Outcomes of maintenance therapy with decitabine and the changes in MRD during this stage are shown in **Table 2** and **Figure 1**. All four patients with MRD-positive disease before maintenance treatment turned negative after two or two cycles. Only three patients (8.8%) had positive MRD once during maintenance therapy. The median time from transplantation to the start of maintenance treatment was 96 days (range, 51–175 days), and the median number of decitabine cycles for all patients was seven (range, 1–14). Overall, 14 patients (41.1%) completed the study and entered the follow-up phase, including 12 patients with 8 cycles, 1 patient with 14 cycles, and 1 patient with 13 cycles of treatment. Patients No. 5 and No. 6 received more than eight cycles because they were MRD positive after the completion of eight cycles of maintenance treatment, and their MRD turned negative after an additional cycle of decitabine (**Figure 1**). At the data cut-off point, eight patients (23.5%) were in the maintenance phase, including six patients who entered the study later and two patients due to the delay caused by the coronavirus disease 2019 (COVID-19) epidemic. The reasons for discontinuation included relapse (n = 4, 11.7%), GVHD (n = 3, 8.8%), and withdrawn consent (n = 5, 14.7%) (**Table 2**). Besides, as shown in **Table 3**, seven patients with Ph^+^ ALL were treated with TKI maintenance during pre-transplantation chemotherapy, conditioning regimen, and post-transplantation maintenance therapy. Notably, TKI was suspended temporarily to reduce the risk of infection in patients with neutropenia after chemotherapy or transplantation.

**Table 3:** Use of TKI in 7 Patients with Ph^+^ ALL.

PatientNo.	TKI Before HSCT	TKI in Conditioning	TKI after HSCT			Relapse
			Starting Time of TKI (days)	Usage of TKI	Time of TKI Withdrawal (days)	
16	Dasatinib (100 mg/d) + chemotherapy	Dasatinib (100 mg/d)	60	Imatinib (400 mg/d)^*^	276	Yes
17	Imatinib (400 mg/d) + chemotherapy	Imatinib (400 mg/d)	61	Imatinib (400 mg/d)	170	Yes
18	Dasatinib (100 mg/d) + chemotherapy	Dasatinib (100 mg/d)	59	Dasatinib (100 mg/d)	223	Yes
19	Imatinib (400 mg/d) + chemotherapy	Imatinib (400 mg/d)	65	Imatinib (400 mg/d)	365	No
20	Imatinib (400 mg/d) + chemotherapy	Imatinib (400 mg/d)	54	Imatinib (400 mg/d)	379	No
21	Dasatinib (100 mg/d) + chemotherapy	Dasatinib (100 mg/d)	66	Dasatinib (100 mg/d)	156	No
			157	Imatinib (400 mg/d)^#^	365	
22	Imatinib (300 mg/d)^§^ + chemotherapy	Imatinib (300 mg/d)	57	Dasatinib (100 mg/d)	366	No

TKI, tyrosine kinase inhibitor; ALL, acute lymphoblastic leukemia; HSCT, hematopoietic stem cell transplantation.

*As the patient suffered from diabetes mellitus complicated with fundus disease, dasatinib was replaced with imatinib after transplantation. ^#^Dasatinib was replaced with imatinib at 157 days after transplantation because of repeated pleural effusion after taking dasatinib.

^§^The patient was intolerant to imatinib (400 mg/d), accompanied by severe nausea and vomiting, so imatinib was reduced to 300 mg/d.

### Relapse

**Figure 2:**
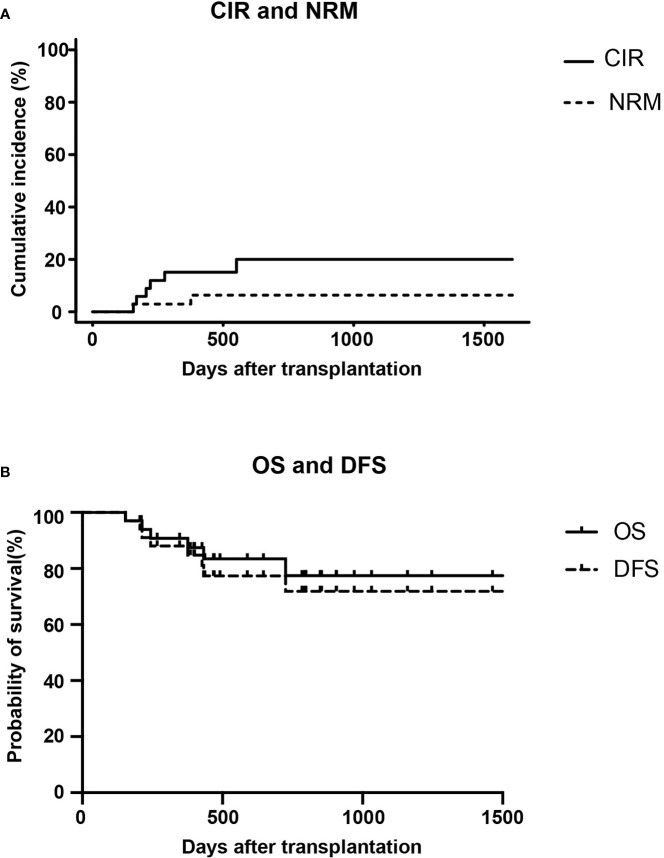
CIR and NRM **(A)** and OS and DFS **(B)** for all patients (n = 34) after allo-HSCT.

**Figure 3:**
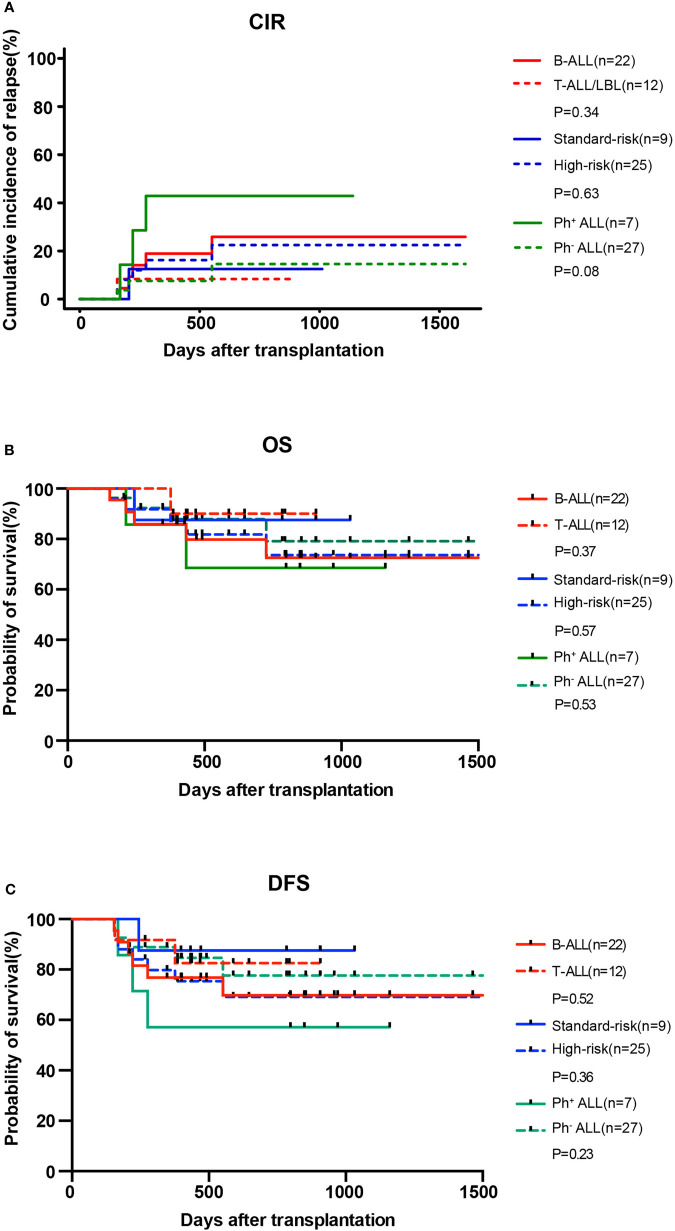
CIR **(A)** and OS **(B)** and DFS **(C)** for patients with B-ALL (n = 22) and T-ALL/LBL (n = 12), patients at high risk (n = 25) and standard risk (n = 9), and patients with Ph^+^ ALL (n = 7) and Ph^−^ ALL (n = 27) after allo-HSCT.

At the data cut-off point (July 2020), the median follow-up time was 480.5 days (range, 154–1629 days) (**Table 2**). A total of six patients relapsed (17.6%) with a median relapse time of 213 days (range, 156–551 days) after transplantation. Of these, five patients were at high risk (three patients with Ph^+^ B-ALL, two of which had *T315I* mutation at the time of relapse; one patient with pro-B-ALL; one patient with T-LBL in the leukemic phase had a WBC count >100×10^9^/L at diagnosis), and one patient with B-ALL was at standard risk (**Table 4**). Among the 14 patients who completed the study, only patient No. 2 presented extramedullary recurrence at 551 days after transplantation. Patient No. 16 with Ph^+^ ALL only took imatinib but stopped decitabine on his own after five cycles. Relapse happened and *T315I* mutation was detected 2 months later, whereas this patient did not take ponatinib due to economic reasons. Patient No. 17 with Ph^+^ ALL relapsed for a second time, and the *T315I* mutation was detected after two cycles. Patient No. 18 with Ph^+^ ALL relapsed after five cycles and refused to test for mutations in the ABL kinase domain. Patients No. 3 with CR2 at HSCT relapsed after three cycles. After the six relapsed patients, one patient received chemotherapy; one patient received chemotherapy, TBI, and DLI in turn; two patients received chemotherapy; and two patients were discharged automatically. Finally, four patients died after relapse, and two patients were still alive (**Table 4**). Interestingly, none of the seven patients with T-ALL relapsed, including one patient with early T-cell precursor ALL (ETP-ALL) and three patients at high risk. In the end, the 2-year CIR of all 34 patients was 20.0%, and the median CIR time was not reached (**Figure 2A**). Patients with T-ALL/LBL and B-ALL had a 2-year CIR of 8.3 and 25.8%, respectively (P = 0.34). Patients at high risk and standard risk had a 2-year CIR of 22.4 and 12.5%, respectively (P = 0.63). Patients with Ph^+^ ALL and Ph^−^ ALL had a 2-year CIR of 42.8 and 14.5%, respectively (P = 0.08) (**Figure 3A**).

**Table 4:** Characteristics and outcomes of six patients with relapse.

Patient No.	Diagnosis	High-Risk Factor at Diagnosis	Disease Status at HSCT	Starting Time of Decitabine (days)	Cycles of Decitabine	Reason for Discontinuation of Decitabine	Bone Marrow Results at Relapse	Time From HSCT to Relapse (days)	Treatment After Relapse	Overall Survival (days)
16	B-ALL	Ph^+^ WBC >100×10^9^/L	CR1	63	5	Withdrew consent	Marrow blast 97.6% *T315I* mutation	276	DLI + chemotherapy	433
2	B-ALL	CD10^−^	CR1	97	8	Completed study	Extramedullary	551	Chemotherapy; TBI; DLI	725
17	B-ALL	Ph^+^ Age >35	CR2	110	2	Relapse	Marrow blast 42% *T315I* mutation	168	Automatic discharge	213
3	B-ALL	No	CR2	84	3	Relapse	Marrow blast 49.6%	205	Automatic discharge	244
30	T-LBL	Leukemic phase; WBC>100×10^9^/L	CR1	93	3	Relapse	Extramedullary	156	Chemotherapy	>205
18	B-ALL	Ph^+^	CR1	60	5	Relapse	Marrow blast 16.4%	221	Chemotherapy	>427

WBC, white blood cell; CR, complete remission; DLI, donor lymphocyte infusion; HSCT, hematopoietic stem cell transplantation.

### DFS and OS

At the data cut-off point, 28 (82.3%) of the 34 patients were alive (82.3%), and 26 patients (76.5%) were alive without relapse/progression. Causes of death included relapse (n = 4), severe infection (n = 1), and GVHD (n = 1) (**Table 2**). The 2-year NRM was 6.3% (**Figure 2A**). The 2-year OS was 77.5%, and the 2-year DFS rate was 73.6% for the 34 patients (**Figure 2B**). The 2-year OS of patients with T-ALL/LBL and B-ALL were 90 and 72.5%, respectively (P = 0.37). The 2-year OS of patients at high risk and standard risk were 73.6 and 87.5%, respectively (P = 0.57). The 2-year OS of patients with Ph^+^ ALL and Ph^−^ ALL were 68.6 and 79.1%, respectively (P = 0.53) (**Figure 3B**). For patients with T-ALL/LBL and B-ALL, the 2-year DFS were 81.5 and 69.6%, respectively (P = 0.52). For patients at high risk and standard risk, the 2-year DFS were 68.8 and 87.5%, respectively (P = 0.36). For patients with Ph^+^ ALL and Ph^−^ ALL, the 2-year DFS were 57.1 and 77.3%, respectively (P = 0.23) (**Figure 3C**).

### GVHD

One patient (2.9%) developed grade IV aGVHD, and seven (20.5%) patients developed cGVHD (three with mild cGVHD and four severe cGVHD) during maintenance treatment phase (**Table 2**). Among the eight patients with GVHD after maintenance treatment, three patients had reduced the dose of immunosuppressive drugs before developing GVHD (one withdrew immunosuppressants and developed grade IV aGVHD during the second cycle; then, the patient stopped using decitabine and received intensive immunosuppressive treatment, but the response was poor and the patient eventually died). Two patients presented cGVHD before maintenance treatment. Of the remaining three patients, two developed cGVHD after one cycle and one developed cGVHD after three cycles of maintenance treatment. Among the eight patients with GVHD, organ involvement included the skin in eight patients, the intestinal tract in two patients, the liver in three patients, the oral cavity in three patients, and the eye in one patient. No significant worsening or relief was observed in patients with GVHD due to the use of decitabine.

### Adverse Events

The main adverse event caused by low-dose decitabine was hematological toxicity. Among the 34 patients, 32 (94.1%) developed grade I to II myelosuppression after maintenance treatment with low-dose decitabine (**Table 4**), and no infection occurred after timely administration of G-CSF. Only two patients (5.8%) developed grade III to IV myelosuppression. Patient No. 9 developed degree IV granulocytopenia and mild pulmonary fungal infection after one cycle, which improved after administration of G-CSF and oral voriconazole. Patient No. 22 developed grade III granulocytopenia and mild pulmonary bacterial infection after one cycle, which improved after administration of G-CSF and oral azithromycin. Their granulocytes returned to normal after 14 days and 12 days of treatment, respectively. None of the patients required blood transfusion during the period of myelosuppression, and none of the patients interrupted treatment because of infection.

## Discussion

Disease relapse is a major therapeutic challenge in patients with adult ALL that have undergone allo-HSCT, and treatment options are limited. The risk of relapse-related death in this population was as high as about 30–54% (1–3, 28), leaving preventing post-transplantation relapse necessary. At present, the downregulation of HLA-II molecules on leukemic cells caused by epigenetic silencing (such as the hypermethylation of CIITA) leads to immune escape of leukemic cells, which is a main mechanism of relapse post-transplantation (4, 8). Moreover, abnormalities in DNA methylation are common in ALL (11–13, 29, 30). Therefore, combined with the clear benefits of HAMs in maintenance therapy after AML/MDS transplantation (14, 15), we first evaluated the use of low-dose decitabine as maintenance therapy after allo-HSCT for adult ALL to reduce relapse and improve survival of this population. In this study, we achieved a 2-year CIR (20%) that was lower than that reported in previous studies, and the 2-year OS (77.5%) was satisfactory. Even in the high-risk group, the 2-year OS was 73.6%. To some extent, our data also indicated that maintenance therapy with decitabine may be used as a treatment option to prevent relapse after ALL transplantation.

The prognosis of adult T-ALL is unsatisfactory, with a 5-year OS of only 30–50% (31–33). Furthermore, the prognosis of patients who relapse is poorer, with a reported 5-year OS of 5% (34). Although allo-HSCT has improved the prognosis of this population, there is still a CIR of 12.4% in the low-risk group and 41.2% in the high-risk group (35). However, the 2-year CIR of patients with T-ALL/LBL in our study was only 8.3%, while OS and DFS were as high as 90 and 81.5%, respectively. Surprisingly, none of the patients with T-ALL experienced relapse, which is encouraging. Katagiri et al. (36) also reported that successful maintenance treatment was achieved with azacitidine in a patient diagnosed with myeloid/lymphoid neoplasm with FGFR1 (located on chromosome 8p11.2) rearrangement after allo-HSCT. In addition, ETP-ALL has a higher rate of remission failure and subsequent relapse than typical T-ALL (37). Meng et al. (38) reported that six patients with relapsed/refractory ETP-ALL were treated with decitabine combined with the CAG regimen (aclarubicin, cytarabine, and G-CSF), and five patients achieved CR. In this study, one patient with ETP-ALL initiated maintenance treatment with decitabine and has completed four cycles and is currently well at the date of last follow-up. The above evidence supports the feasibility of low-dose decitabine maintenance therapy in T-ALL.

Lockhart et al. (39) described a child with Ph^+^ ALL having mixed donor chimerism and persistent BCR-ABL transcripts after allo-HSCT. There was no response to TKI treatment, but her clonal cytogenetic abnormalities were resolved after decitabine treatment. Cui et al. (40) also described 12 patients with relapse ALL after transplantation who were treated with decitabine alone or in combination with chemotherapy and DLI, and found that patients with Ph^+^ ALL achieved higher survival than patients with Ph^−^ ALL. However, the effects of decitabine maintenance treatment on patients with Ph^+^ ALL was not significant, and the 2-year CIR was much higher than that of patients with Ph^−^ ALL in this study. Although all seven patients with Ph^+^ ALL received oral TKI after transplantation, three patients still relapsed. However, this may be related to the presence of the *T315I* mutation, as in two of the three relapsed patients the *T315I* mutation was detected at relapse, and one patient was not tested voluntarily. For patients with Ph^+^ ALL after transplantation, exploring treatment with next-generation TKI may be more meaningful than low-dose decitabine.

HMAs can upregulate the expression of FOXP3 in CD4+CD25-T cells, thus increasing the number of Treg cells and mitigating GVHD (41, 42). In this study, only one patient developed grade IV aGVHD, while four patients presented severe cGVHD. Most cases occurred when immunosuppressants were reduced or prior to maintenance treatment. Only three patients stopped maintenance treatment because of GVHD. It is a pity that no aggravation or relief was observed in patients with GVHD due to the use of decitabine.

In this study, 94.1% patients developed grade I–II myelosuppression after receiving low-dose decitabine. Only two patients (5.8%) developed grade III–IV granulocytopenia and mild pulmonary infection. None of the patients required blood transfusion, and no one stopped this trial because of hematological toxicity. Pusic et al. (15) divided 24 patients with AML/MDS into four groups after transplantation, and each group was given different doses of decitabine for maintenance therapy. The authors found that the 10 mg/m^2^/d group presented fewer hematological adverse reactions and that decitabine was well tolerated, which was similar to our results. Further, our study showed that maintenance treatment with low-dose decitabine after transplantation had low hematological toxicity and is well tolerated.

Obviously, this study also has some limitations. First, our patients exhibited selection bias. Risk of disease relapse after allo-HSCT is a composite of multiple factors, including age, risk stratification at diagnosis, remission status at the time of transplantation, and duration of remission after transplantation. This study enrolled patients who were relatively young, and the sample population included 26.9% low-risk patients and several patients who were still in CR about 6 months after transplantation, which would lead to a better overall prognosis. Conversely, patients were enrolled without severe complications such as severe GVHD and were selected after day +50 of HSCT, which necessarily excluded those who relapsed early. Therefore, some transplanted patients were excluded because of early relapse or non-relapse mortality within the first 2 months. Secondly, this study did not detect changes in DNA methylation level before and after treatment, which would further support the use of HAMs. Finally, this study is limited by the small number of patients and lack of controls.

In conclusion, although the current data do not provide definitive evidence supporting the effects of low-dose decitabine maintenance treatment on the prevention of relapse after ALL transplantation, the overall results are encouraging and still indicate a positive trend. Low-dose decitabine maintenance treatment may be used as an option to prevent relapse after transplantation in patients with adult ALL, especially in patients with T-ALL. Our findings require confirmation in larger-scale controlled trials.

## Data Availability Statement

The original contributions presented in the study are included in the article/supplementary material. Further inquiries can be directed to the corresponding authors.

## Ethics Statement

The studies involving human participants were reviewed and approved by the Ethics Committee of The First Affiliated Hospital of Zhengzhou University. Written informed consent to participate in this study was provided by the participants’ legal guardian/next of kin.

## Author Contributions

RG, JL, and Z-XJ conceived, designed, and planned the study. All authors acquired the data. JL analyzed the data. JL and RG interpreted results. JL, FH, and RG drafted the report. RG, R-QL, and WL were involved in the critical revision of the manuscript. All authors contributed to the article and approved the submitted version.

## Funding

This work was supported by the National Natural Science Foundation of China (81070445), Natural Science Foundation of Henan Province (No. 182300410301), Medical Science and Technology Research Project of Henan Province (2018020118), the Key Scientific Research Project of Higher Education of Henan Province (No. 18A320050), Science and Technology Plan of Henan Province (No. 182102310160), the Candidate Project of Henan Provincial Medical Science and Technology Research Funds Jointly Built by Province and Ministry (No. 2018010001).

## Conflict of Interest

The authors declare that the research was conducted in the absence of any commercial or financial relationships that could be construed as a potential conflict of interest.

## Publisher’s Note

All claims expressed in this article are solely those of the authors and do not necessarily represent those of their affiliated organizations, or those of the publisher, the editors and the reviewers. Any product that may be evaluated in this article, or claim that may be made by its manufacturer, is not guaranteed or endorsed by the publisher.
